# Cloning and Functional Characterization of *SAD* Genes in Potato

**DOI:** 10.1371/journal.pone.0122036

**Published:** 2015-03-31

**Authors:** Fei Li, Chun Song Bian, Jian Fei Xu, Wan fu Pang, Jie Liu, Shao Guang Duan, Zun-Guo Lei, Palta Jiwan, Li-Ping Jin

**Affiliations:** 1 The Institute of Vegetables and Flowers, Chinese Academy of Agricultural Sciences, Beijing, China; 2 Institute of Potato in Guizhou Province, Guiyang, China; 3 Department of Horticulture, University of Wisconsin, Madison, Wisconsin, United States of America; Chinese Academy of Sciences, CHINA

## Abstract

Stearoyl-acyl carrier protein desaturase (SAD), locating in the plastid stroma, is an important fatty acid biosynthetic enzyme in higher plants. SAD catalyzes desaturation of stearoyl-ACP to oleyl-ACP and plays a key role in determining the homeostasis between saturated fatty acids and unsaturated fatty acids, which is an important player in cold acclimation in plants. Here, four new full-length cDNA of SADs (*ScoSAD*, *SaSAD*, *ScaSAD* and *StSAD*) were cloned from four *Solanum* species, *Solanum commersonii*, *S*. *acaule*, *S*. *cardiophyllum* and *S*. *tuberosum*, respectively. The ORF of the four SADs were 1182 bp in length, encoding 393 amino acids. A sequence alignment indicated 13 amino acids varied among the SADs of three wild species. Further analysis showed that the freezing tolerance and cold acclimation capacity of *S*. *commersonii* are similar to *S*. *acaule* and their SAD amino acid sequences were identical but differed from that of *S*. *cardiophyllum*, which is sensitive to freezing. Furthermore, the sequence alignments between *StSAD* and *ScoSAD* indicated that only 7 different amino acids at residues were found in SAD of *S*. *tuberosum* (Zhongshu8) against the protein sequence of *ScoSAD*. A phylogenetic analysis showed the three wild potato species had the closest genetic relationship with the SAD of *S*. *lycopersicum* and *Nicotiana tomentosiformis* but not *S*. *tuberosum*. The SAD gene from *S*. *commersonii (ScoSAD)* was cloned into multiple sites of the pBI121 plant binary vector and transformed into the cultivated potato variety Zhongshu 8. A freeze tolerance analysis showed overexpression of the *ScoSAD* gene in transgenic plants significantly enhanced freeze tolerance in cv. Zhongshu 8 and increased their linoleic acid content, suggesting that linoleic acid likely plays a key role in improving freeze tolerance in potato plants. This study provided some new insights into how SAD regulates in the freezing tolerance and cold acclimation in potato.

## Introduction

Following wheat and rice, potato (*Solanum tuberosum* L) is ranked the third most important food crop across the world. However, potato is unable to survive when the temperature dropped to minus 3 degree [1]. Freezing stress therefore is one of the major challenges in potato production in many parts of the world. For example, the direct economic losses over $1.5 billion USD of potato were caused by the ice storm in south China in 2008 [2]. To identify the freeze-tolerant potato germplasms, a large scale screening was performed in various potato species and a number of wild potato species with different freeze tolerance traits were obtained. 30% of them with high freezing tolerance traits were selected as candidates for improving the current cultivars. Of these wild resources, *S*. *commersonii* and *S*. *acaule* are highly tolerant to freezing and have cold acclimation capacity, whereas *S*. *cardiophyllum* is sensitive and has no cold acclimation capacity [1, 3].

It is well known that the unsaturated fatty acids play a crucial role in freeze tolerance in plants. Plants contain both unsaturated and saturated fatty acids. The freezing tolerance of plants largely depends on the homeostasis between unsaturated and saturated fatty acids [4]. Stearoyl-acyl carrier-protein desaturase (SAD) can bridge the lipid transition from saturated fatty acids to unsaturated ones and is the key rate-limiting enzyme in the unsaturated fatty acid biosynthetic pathway. SAD catalyzes dehydrogenation of the stearoyl acyl carrier protein (18:0-ACP) and thus generates the oleoyl-acyl carrier protein by introducing the first double bond between carbon atoms 9 and 10 in the fatty acid chain. Oleoyl-acyl carrier protein is a substrate of polyunsaturated fatty acids, such as linoleic acid (C18:2) and linolenic acid (C18:3) [5, 6].

In 1989, Abdallah and Palta [7] reported that the content of C18:2 could be enhanced in plants through cold acclimation. C18:2 is a key player to improve plant a freeze tolerance. Seasonal variation of C18:2 content has also been observed in pine needles [8], cranberry [7] and rice [9], suggesting that freeze tolerance is tightly correlated with C18:2 content. Introduction of the first double bond into cell membrane fatty acids is essential for conversion from a gelatinous to a crystalline state [10]. SAD plays a key role in determining the ratio of saturated to unsaturated fatty acids. Thus, the formation, maintenance, optimal mobility and semi permeability of the cell membranes in plants are largely regulated by SAD [11]. Several observations showed that the freeze tolerance of plants can be enhanced to some extent through increasing the expression of *SAD* [12, 13, 14].

*SAD* genes have been isolated from various plants including cucumber, castor bean [15], sunflower [6], spinach [16] and bird rape [17]. Additionally, freezing-related genes (such as *SM2358*, *MasCIC*, *A13*) and freezing-inducible genes (e.g. *CI7*, *CI13*, *CI19* and *CI21*) have been cloned from several potato species [18, 19, 20]. Moreover, using the freeze-tolerant wild potato, *S*. *sogarandinum*, Roratet et al. [21] isolated the *Ssci1*, *Ssci12*, *Ssci17*, and *Ssci20* genes, which were upregulated in *S*. *sogarandinum* compared to the freeze-sensitive cultivar *S*. *tuberosum* during freeze treatment. However, the amino acid sequence variation in the *SAD* genes between freeze-tolerant and-sensitive wild potato species has not been reported. In this study, we cloned the *SAD* genes from freeze-tolerant potato species, *S*. *commersonii* and *S*. *acaule*, the freeze-sensitive wild potato species *S*. *cardiophyllum and S*. *tuberosum*, respectively. The functions of *SAD* genes have been characterized through introducing them into common potato cultivars. Several independent transgenic lines have been obtained and found the freeze tolerance of potato tightly correlated with the expression levels of the SADs. Our observations provide some new insights into improving freeze tolerance in potato.

## Materials and Methods

Three wild potato species, *S*. *commersonii* (PI 473412), *S*. *acaule* (PI 472678), and *S*. *cardiophyllum* (PI 341233), were obtained from the Inter Regional Potato Introduction Station (NRSP-6), Sturgeon Bay, WI, USA. The materials were cultivated in 10 × 10-cm plastic pots containing vermiculite and turf soil mixed substrate (1:1) in March 2012. Nine plants of each species were cultivated in pots and incubated in an artificial incubator at a temperature of 22 ± 1°C, illumination intensity of 400 μmol m^–2^ s^–1^, photoperiod of 14 h/10 h, and relative humidity of 70 ± 5%. The healthy minitubers of Zhongshu8 were sliced into longitudinal sections which were used as explants for genetic transformation.

### Assays for cold acclimation and freezing tolerance in potato

The cold treatment of potato was initiated 5 weeks after planting. To achieve cold acclimation, the temperature inside the incubator was lowered to 4°C/2°C (day/night) with a 14-h photoperiod and 100 μmol m^−2^ s^−1^ of photosynthetically active radiation for 12 d. The freezing tolerance of potato was examined as described by Vega [22]. Fully expanded terminal leaflets were excised at the end of the dark period and placed in covered glass culture tubes. The glass tubes, containing one leaflet each, were submerged in glycol in a temperature-controlled cooling bath (Forma Scientific, Model 2323, Marietta, OH, USA). The cooling rate: above −10°C, was 0.5°C/30 min; and below −10°C was 1.0°C/30 min. Ice nucleation was initiated at −1.0°C by adding a small piece of ice to each tube. Tubes containing frozen leaflets were removed at predetermined temperatures (used to develop an ion leakage curve) and thawed on ice overnight prior to injury evaluation. Leaflets used as unfrozen controls were stored in an ice-filled cooler. Three leaflets per potato species were evaluated before and after cold acclimation at each temperature.

Freeze injury was assessed by measuring ion leakage [23]. Ion leakage was expressed as the ratio of electrolyte leakage from freeze-injured tissue to that from autoclaved tissue. Thawed leaflets were sliced into strips before adding 25-ml deionized distilled water, and then infiltrated for 5 min at 10 kPa using a vacuum pump, and shaken for 1 h at 220 rpm on a gyratory shaker at room temperature. Electrical conductivity (R1) was measured with an YSI model 32 conductance meter (Yellow Springs Instruments, Yellow Springs, OH, USA). The total conductivity (R2) of each sample was measured following a 24h cooling period after autoclaving at 121°C for 15 min. Percent mean ion leakage was expressed as (R1/R2) × 100 in triplicates and was plotted as a function of freezing temperature. Relative freeze tolerance was determined from the midpoint of the maximum (autoclaved) and minimum (control) ion leakage values obtained for each potato species (average of three leaflets) before and after cold acclimation, as described by Vega et al. [22].The absolute value of this temperature was defined as the RFT[24]. Acclimation capacity (ΔRFT) was expressed as acclimated RFT minus nonacclimated RFT.

#### RNA isolation and first-strand cDNA synthesis

Total RNA was extracted from plants using TRIzol reagent (Invitrogen, Carlsbad, CA, USA), and cDNA was synthesized using SuperScript III First Strand Kits (Invitrogen, Carlsbad, CA, USA), according to the manufacturer’s instructions.

### Primer design and synthesis

Degenerate primers were designed based on the conserved region of the *SAD* gene sequence (GenBank accession no. X78935.2) using Primer Premier 5. The primers sequence F101 and R1419 are listed in Table 1.

**Table 1:** Sequences of primers.

Primer name	Primer sequences (5’-3’)	Product length (bp)
F101	ATCTGTCTCGAAGCCTCTTC	1318
R1419	CCTATGGTGATTGGTGTTCC	
F705	TACAGCTCGGCATGCTAA	519
R29	GGGTTGGGGTTTCTACAG	
ProF	GAGAGGCTTACGCAGCAGGT	596
ProR	GGCAGAGGCATCTTCAACGA	
β-tubulin2-F	GATGTTGTGCCAAAGGATGT	221
β-tubulin2-R	AACTTGTGGTCAATGCGAGA	
QsadF	GTCCGTGTTCACCACTCAGA	137
QsadR	GCGGCATGGAATGAGTAACT	

### Reverse transcription-polymerase chain reaction (RT-PCR)

Degenerate primers were used for PCR amplification with the first-strand cDNA as the template. The PCR system contained 4-μl 5× PCR buffer, 0.8-μl 5 mM dNTPs, 0.8-μl 5 μM forward primer, 0.8-μl 5 μM reverse primer, 0.2-μl GO Taq DNA polymerase, 2-μl 30 ng/μl cDNA template, and ddH_2_O to a final volume of 25 μl. PCR was initiated for 5 min at 95°C for predenaturation, followed by 35 cycles of 95°C for 30 s, 60°C for 1 min, 72°C for 2 min, and a final extension at 72°C for 7 min. The PCR product was electrophoresed in a 1.2% agarose gel and the purified band was ligated into the pGEM-T vector and transformed into competent *E*. *coli* DH5α cells, which were plated on white–blue selective plates, and positive clones were identified by colony PCR and sequencing.

### Sequence alignment and structural prediction

The open reading frame of the *SAD* gene was deduced using the Bioxm 2.6 software. The amino acid sequences were aligned using the NCBI Blast software. A phylogenetic tree was constructed using Clustal 1.8.1 and Mega 5.05. Multiple sequence alignment was conducted using the DNAMAN 5.0 software. The protein domain analysis was performed using Pfam 27.0 (http://pfam.sanger.ac.uk/).

### Construction of the expression vector

The *SAD* gene from *S*. *commersonii* cDNA was cloned and designated *ScoSAD*. A full-length fragment (1182 bp) of the *ScoSAD* cDNA was inserted into the pBI121 (AF485783) binary vector [25] with the CaMV35S promoter upstream and the selectable neomycin phosphotransferase II marker gene downstream. Then, the construct carrying the *ScoSAD* coding sequence was introduced into *Agrobacterium tumefaciens* by electroporation method.

### *Agrobacterium*-mediated *ScoSAD* gene transformation

The transformation was performed according to the method of Lu et al. [26]. Just-budding mini-tubers (*S*. *tuberosum* cv. Zhongshu8) were inoculated with *A*. *tumefaciens* strain EHA 105 containing the *35S*:*ScoSAD* plasmid on MS medium (4 mg/l ZT and 0.2 mg/l indole-3-acetic acid [IAA]) and incubated at 28°C in the dark for 2 d. Then, the explants were transferred to MS medium containing 4 mg/l ZT, 0.2 mg/L IAA, and 250 mg/l TIM for 7 d. The rooted transgenic plantlets were selected by means of kanamycin resistance, and further screened by PCR for the *SAD* genes.

### Molecular verification of transformants

PCR primers were designed according to the nucleotide sequence of the *SAD* and *GUS* genes in *pBI121*:*ScoSAD*, spanning nucleotide 705 of the *ScoSAD* gene to nucleotide 29 of the *GUS* gene, which generated a 519-bp fragment. The primers sequence of F705 and R29 are listed in Table 1. The optimal annealing temperature was 60°C via touchdown PCR.

The PCR reaction comprised 5 μl 5× PCR mix, 0.5 μL 10 μM forward primer, 0.5 μl 10 μM reverse primer, 1 μl 30 ng/μl cDNA template, and ddH_2_O to a final volume of 10 μl. PCR was initiated for 3 min at 95°C for predenaturation, followed by 35 cycles of 95°C for 30 s, 60°C for 30s, 72°C for 30s, and a 7 min final extension at 72°C. The PCR product was detected via electrophoresis in a 1.2% agarose gel.

The probe primers were designed based on the CaMV35S promoter sequence using Primer Premier 5, and generated a 596 bp fragment named *Pro-35S*. The primers sequence of ProF and ProR are listed in Table 1. The probe was DIG labeled via PCR. Thirty micrograms of genomic DNA from the transgenic and non-transgenic plants were digested with *Eco*RI-HF.

Total RNA was extracted from leaves on days 0 and 12 after cold treatment and reverse-transcribed to first-strand cDNA using kits (Invitrogen). qRT-PCR was performed using cDNA as the template with an ABI StepOne Real-time PCR System. The PCR reaction comprised 10 μl 2× TransStar Green qPCR Super Mix, 0.4μl Passive Reference Dye, 2.0 μl cDNA, 0.4 μl of 10 μmol L^−1^ QsadF and QsadR, and 6.8-μl ddH_2_O. The reaction program was 95°C for 30 s for predenaturation, followed by 40 cycles of 95°C for 15s, 60°C for 15 s, and 72°C for 10s. Each treatment comprised three biological replicates. The qPCR primers (QsadF and QsadR) and internal reference genes(β-tubulin2-F and β-tubulin2-R) are listed in Table 1.

### Freeze tolerance appraisal of transgenic plants

The appraisal method was the same as that described above by Vega. The freeze tolerance of transgenic plants was evaluated before and after cold acclimation. The difference between the two evaluations was therefore considered the acclimation capacity of the transgenic plants. Cold acclimation capacity (ΔRFT) was assessed as acclimated RFT minus non-acclimated RFT.

### Fatty acid composition analysis of transgenic plants

Powdered potato leaves (0.5 g), treated by cryodesiccation at −70°C, were mixed with 10 ml light petroleum and ultrasound-extracted for 40 min for gas chromatography–mass spectroscopy (GC-MS). The light petroleum was removed via rotary evaporation. Each sample comprised three parallel replicates. The extract was mixed with 1 ml 2.5 M sulfuric acid methanol solution and incubated in a 70°C water bath for 30 min. Two-milliliters n-hexane were added to collect the methyl esterified products; 1 ml n-hexane was used for the second extraction. The two extracts were mixed, a few drops of anhydrous sodium sulfate were added, the solution was centrifuged at 7000 rpm for 5 min, and the supernatant was transferred to a 2 ml centrifuge tube for testing.

The GC-MS conditions were: chromatic column, VF-WAX (30 m × 0.25 mm, 0.25 μm); column oven temperature, 100°C, injection temperature, 250°C; sampling time, 1 min; carrier gas, He; pressure, 73 kPa; total flow, 4 ml/min; column flow, 1 ml/min; linear velocity, 37.2 cm/sec; purge flow, 3 ml/min; ion source temperature, 220°C; interface temperature, 220°C; and solvent cut time, 4 min.

The experimental data were processed using the Xcalibur software. The unknown compounds were analyzed by blasting in NIST, together with the artificial spectrum index, and matching the NIST and Wiley spectral libraries. The percentage of each chemical compound in the samples was calculated using the peak area normalization method.

## Results

### The freeze tolerance appraisal showed that *S*. *commersonii* and *S*. *acaule* displayed the traits of freezing tolerance

To evaluate the freeze tolerance of potato, three wild potato species were selected to perform the RFT assay. At the beginning of cold treatment (0 day), *S*. *acaule* showed the lowest RFT, followed by *S*. *commersonii* and *S*. *cardiophyllum*. After 12 days, the RFT of *S*. *commersonii* dropped to—10.1 ± 0.31 aA while the RFT of *S*. *acaule* and *S*. *cardiophyllum* are—8.9 ± 0.90 bA and—2.3 ± 0.42 cB, respectively (Table 2). The cold acclimation capacity indicated by ΔRFT reached to a very significant level in *S*. *cardiophyllum* and *S*. *acaule* while the reduction of RFT in *S*. *cardiophyllum* is not significantly reduced after cold treatment for 12 days, suggesting that the freeze tolerance of these two species was significantly enhanced by cold acclimation. Since *S*. *commersonii* and *S*. *acaule* are freeze-tolerance and *S*. *cardiophyllum* is freeze-sensitive, our data obtained from RFT assay are highly consistent with their genetic phenotypes. These observations likely reflected that the freeze-tolerant potato species contain more acclimation capacity than that of freeze-sensitive species.

**Table 2:** Nonacclimated freezing tolerance (RFT) and acclimation capacity (ΔRFT), and acclimated freezing tolerance at 4°C day/2°C night for three *Solanumm* species.

*Solanum* species	Noacclimated freezing tolerance	Acclimated freezing tolerance	Acclimation capacity
	(RFT in °C)	(RFT in °C)	(ΔRFT in °C)
*S*. *commersonii*	–3.4 ± 0.20 bB	–10.1 ± 0.31 aA	6.8 ± 0.50 aA
*S*. *acaule*	–5.3 ± 0.08 aA	–8.9 ± 0.90 bA	3.5 ± 1.15 bB
*S*. *cardiophyllum*	–1.8 ± 0.07 cC	–2.3 ± 0.42 cB	0.5 ± 0.36 cC

Acclimation capacity (ΔRFT) was assessed as acclimated RFT minus nonacclimated RFT.

RFT was determined from the midpoint of the maximum and minimum (control) ion leakage values obtainied from each species (24)

Standard error calculated from three experiments. Data are means plus standard errors. Values followed by different letters are significantly different at P ≤ 0.01 (capital letters) or P ≤ 0.05 (small letters).

### Molecular characterization of *SAD* genes in different *Solanum* species

To further dissect the underlying molecular mechanism of the freeze-tolerance and sensitive potato species, we therefore cloned the *SAD* genes, which are the key players in plant cold acclimation, from these three wild species and *S*. *tuberosum* (cv Zhongshu8). RT-PCR was performed to get the SAD genes by using the F101 and R1419 primer pairs listed in Table 1. The ~1300 bp PCR products containing the putative *SAD* genes were obtained from four different *Solanum* species. Further sequence analysis showed that all the four *SAD* genes contain a similar ORF, encoding a putative SAD protein with 393 amino acids. The amino acid sequence identity of the four *SAD* genes was as high as 98.98%, and contained two conserved ferritin-like and acyl-ACP desaturase family domains. We deposited their sequences into GenBank with accession numbers JX412961, JX412962, JX412963 and KP671494.

**Fig 1:**
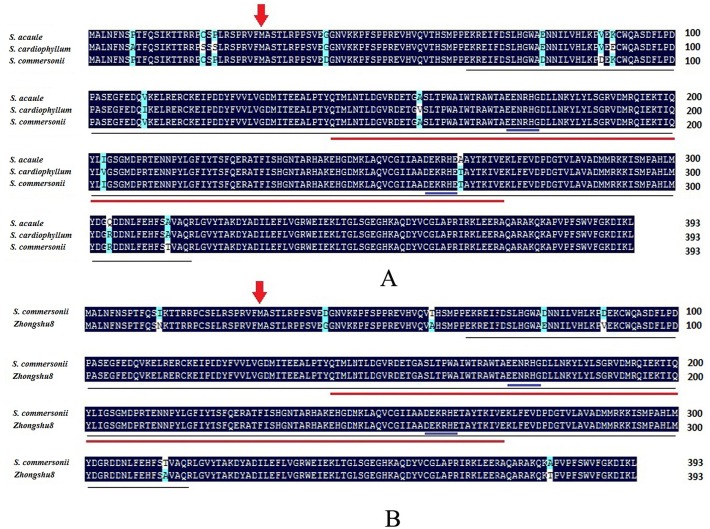
Alignment amino acid sequences of stearoyl-acyl carrier protein desaturases (SAD) of different potato species, including *S. acaule*, *S. cardiophyllum* and *S. commersonii* (A), *S. commersonii* and *S.tuberosum*, cv Zhongshu8 (B). Red arrow indicates the putative cleavage site of the chloroplast-transit peptide, and blue underlined sequence indicates the conserved iron-binding sites of the catalytic domain. The acyl-ACP desaturase and ferritin-like family domains are underlined in black and red, respectively.

The sequence alignments indicated that 7 out of 13 amino acids differed between the freeze-tolerant and freeze-sensitive wild potato species at residues 6, 19, 21, 89, 109, 156, and 202 (Fig 1A). The SAD amino acid sequences of *S*. *commersonii* (No. JX412961) and *S*. *acaule* (JX412962) were identical but different to that of *S*. *cardiophyllum* (JX412963). Amino acid residues 89, 109, 156, and 202 were located in the acyl-ACP desaturase domain. These variations in the *SAD* gene likely affected the cold acclimation capacity of *S*. *cardiophyllum*. The conserved domains were identical among the three *SAD* genes (underlined region in Fig 1A). Amino acid residues 40, 77, 87, and 313 of the *S*. *acaule SAD* gene were identical to those of the SAD from the freeze-sensitive species *S*. *cardiophyllum* but different from that of the freeze-tolerant species *S*. *commersonii*. The amino acid residues 264 and 303 of the *S*. *cardiophyllum SAD* gene were identical to those of the *S*. *commersonii SAD* gene but different from those of *S*. *acaule*. Therefore, the variations in these six amino acid residues were probably not related to cold acclimation. On the other hand, the sequence alignments between *StSAD* and *ScoSAD* indicated that only 7 different amino acids at residues 12, 40,58,77,87,313 and 378 were found in SAD of *S*. *tuberosum* (Zhongshu8) against the protein sequence of *ScoSAD* (Fig 1B), suggesting that the high sequence conservatism of *SAD* protein was observed in different potato species.

### SAD genes showed evolutionary conservation and diversification among various plant species

**Fig 2:**
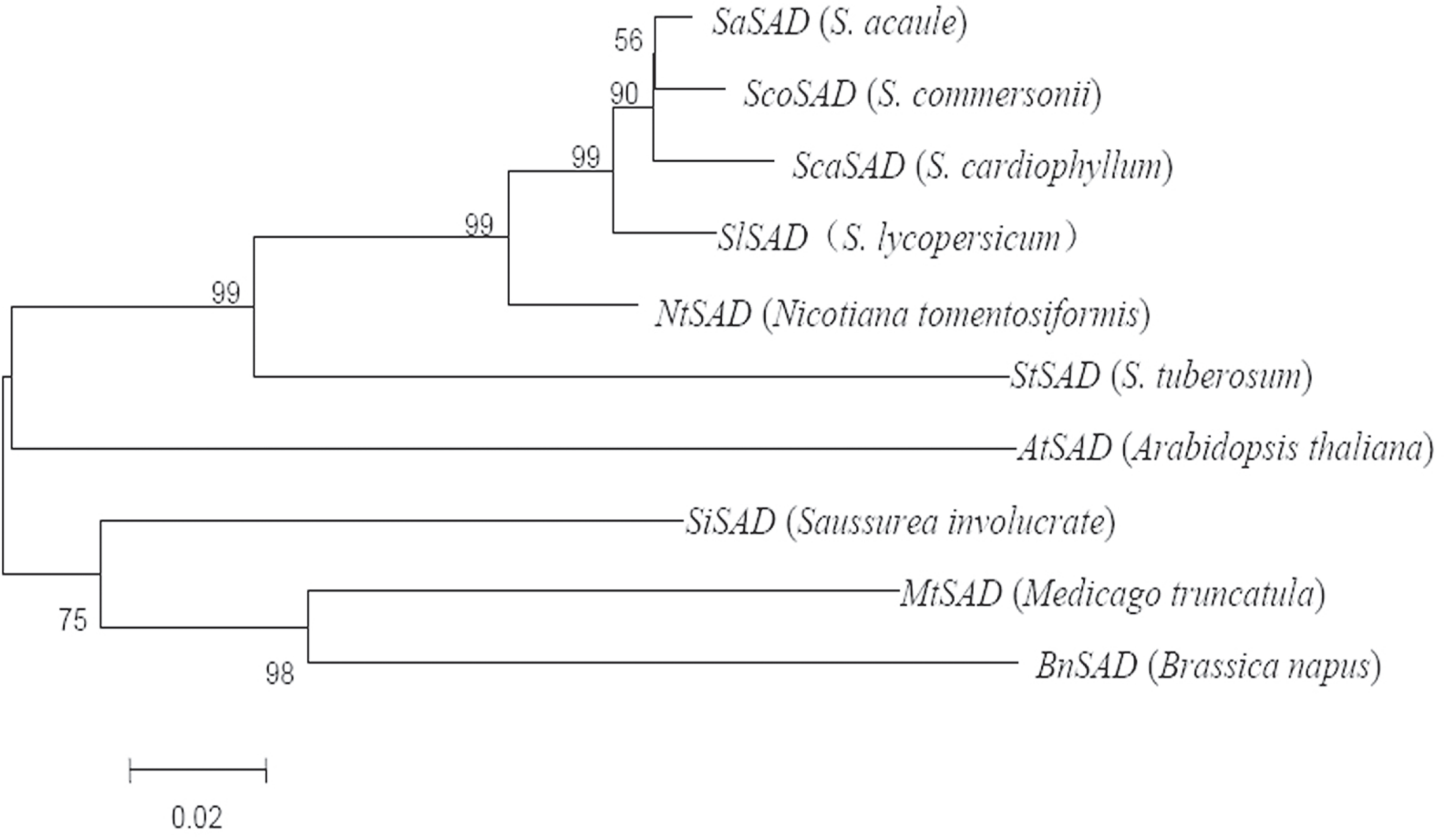
Neighbor-joining tree of the stearoyl-acyl carrier protein desaturase (SAD) amino acid sequences of 10 species.

Although *SAD* genes showed evolutionary conservation and functional diversification in three wild potatoes and one cultivar, the phylogenetic relationships among diverse plant species remain elusive. The protein sequences of SAD homologs from different plant species, including *S*. *acaule*, *S*. *commersonii*, *S*. *cardiophyllum*, *S*. *lycopersicum*, *Nicotiana tomentosiformis*, *S*. *tuberosum*, *Arabidopsis thaliana*, *Saussurea involucrate*, *Medicago truncatula* and *Brassica napus*, were collected for phylogenetic analysis by using Clustal 1.8.1. A neighbor-joining phylogenetic tree based on the SAD amino acid sequences was constructed using MEGA 4.1 (Fig 2). These ten SAD homologs can be clustered into 2 subgroups. It is surprising to observe that the SADs from three wild potato species showed the closest evolutionary relationship with the SAD of *S*. *lycopersicum* and *Nicotiana tomentosiformis* but not *S*. *tuberosum*. The genes from *Saussurea involucrate*, *Medicago truncatula* and *Brassica napus* were clustered into another group. This result indicated SADs show evolutionary conservation and functional diversification in *Solanum* species.

### 15 transgenic potato plants containing *P35S*::*ScoSAD* were obtained

**Fig 3:**
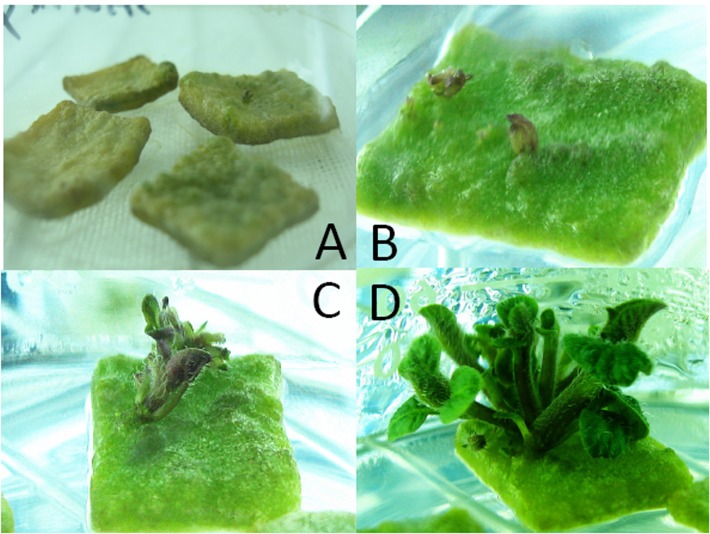
Process of buds induction from potato tuber dish A: Nov.26th, 2012; B: Dec.1th, 2012; C: Dec.5th, 2012; D: Dec.10th, 2012.

The full-length ORF (1182 bp) of the *ScoSAD* was sub-cloned into T-vector and verified by DNA sequencing. *ScoSAD* was further digested by Xba I and Sma I and then inserted it into the pBI121 (AF485783) binary vector through using Xba I and Sma I restriction enzyme site. The destination vector showed that *ScoSAD* was driven by CaMV35S promoter and followed by the selectable neomycin phosphotransferase II marker gene for selection marker in agrobacteria transformation. Then, the resulting construct carrying the *ScoSAD* coding sequence was introduced into *Agrobacterium tumefaciens* by electroporation method. Mini-tubers (*Solanum tuberosum* cv. Zhongshu8) were inoculated with *A*. *tumefaciens* strain EHA 105 containing the *35S*:*ScoSAD* plasmid on MS medium (4 mg/l ZT and 0.2 mg/l indole-3-acetic acid [IAA]) and incubated at 28°C in the dark for 2 d. Then, the explants were transferred to MS medium containing 4 mg/l ZT, 0.2 mg/L IAA, and 250 mg/l TIM for 7 d (Fig 3). The rooted transgenic plantlets were selected by means of kanamycin resistance, and further screened by PCR for the *SAD* genes. Of the 35 regenerated plants, 15 (42.8%) were PCR positive. A ~500-bp fragment was amplified from the positive control *pBI121*:*ScoSAD* plasmid and some of the transgenic plants, but not the non-transgenic plants. These plants were transferred to an illuminating incubator under the condition that temperature is 22 ± 1°C, illumination intensity is 400 μmol m^–2^ s^–1^, photoperiod is 14 h/10 h, and relative humidity is 70 ± 5%.

### Molecular characteration of *P35S*::*ScoSAD* transgenic plants

**Fig 4:**
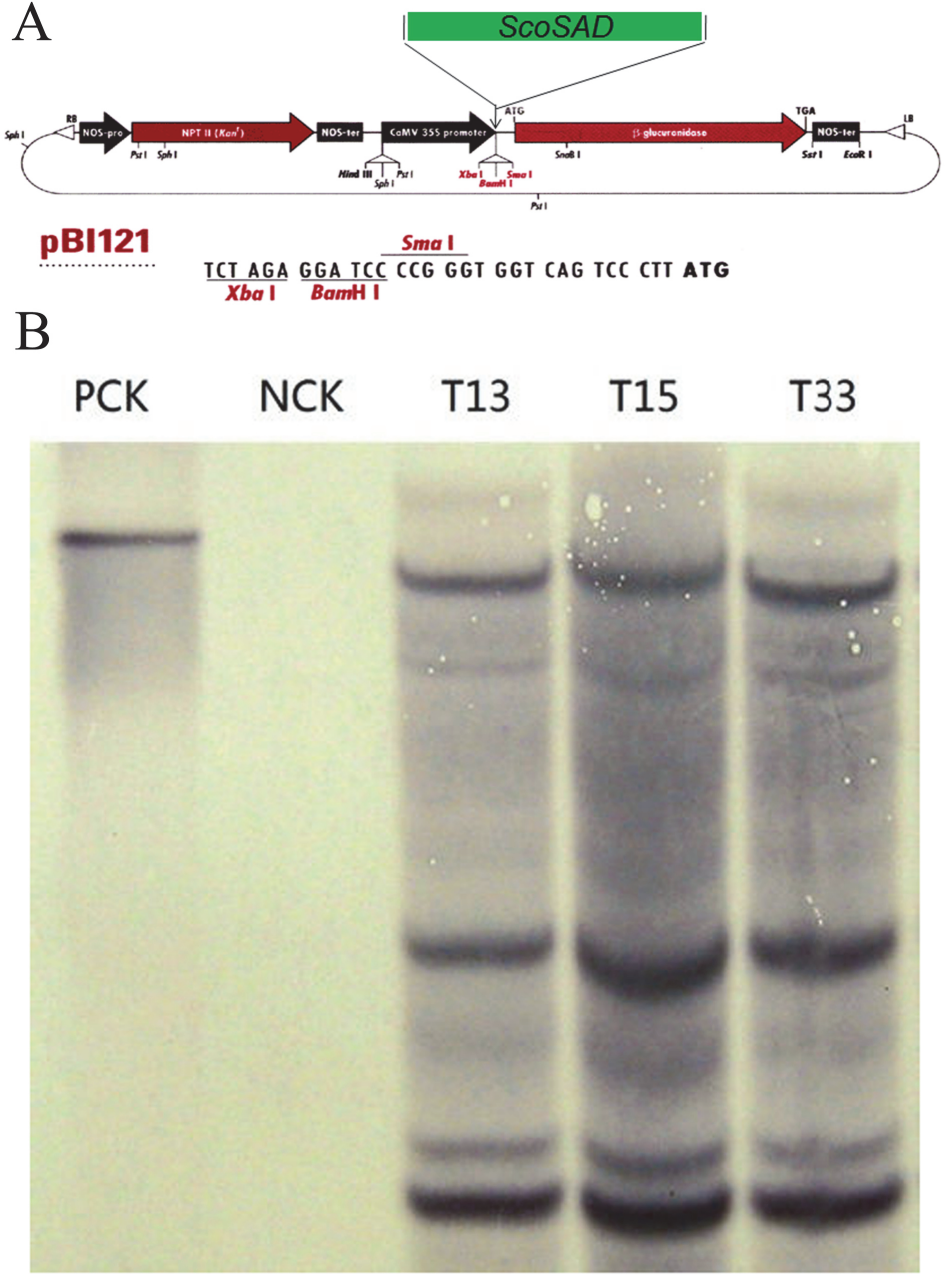
The pBI121 binary vector (A) and Southern blots of transgenic plants harboring the stearoyl-acyl carrier protein desaturase gene from *S*. *commersonii* (*ScoSAD*) (B). PCK, *pBI121*:*ScoSAD* plasmid; NCK, wild-type Zhongshu8 plant; T13-T33, *ScoSAD*-overexpressing lines.

**Fig 5:**
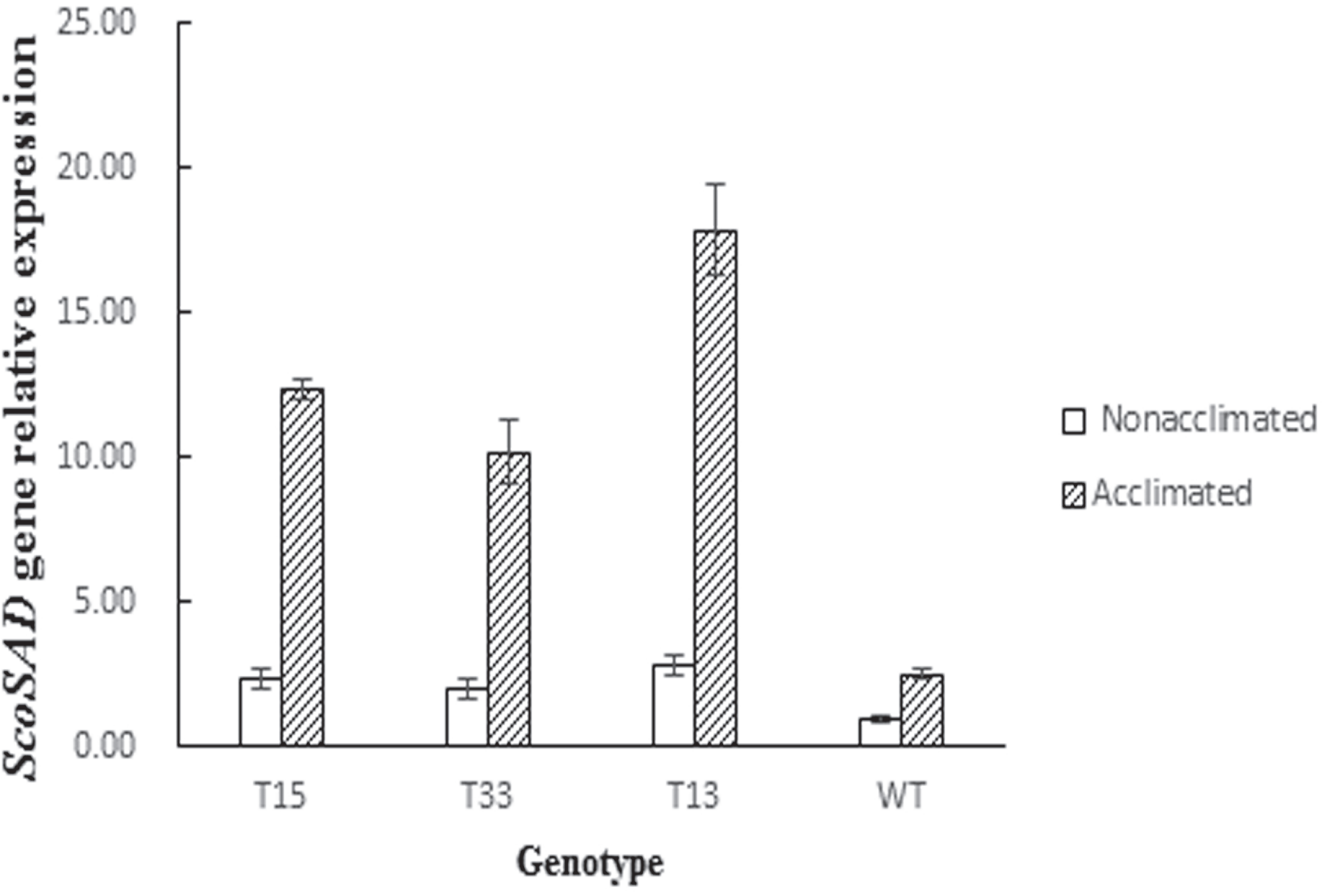
Expression of the stearoyl-acyl carrier protein desaturase (SAD) gene in four potato genotypes following cold acclimation.

To further confirm the putative transgenic potato plants really contain the exogenous *ScoSAD*(Fig 4A), genomic DNA were extracted from the putative transgenic plants (T13, T15, and T33) according to CTAB method, and southern blot was carried out to examine whether the exogenous *ScoSAD* was stably integrated into the genome of *S*. *tuberosum* (Zhongshu8). The Fig 4B showed that the plasmid-control *pBI121*:*ScoSAD* and the *ScoSAD*-overexpressing lines T13, T15, and T33 displayed clear hybridization signals but did not occur in Zhongshu8(WT control). This suggests the exogenous *ScoSAD* gene was stably integrated into the genome of Zhongshu8. Furthermore, Real-time PCR was employed to quantitatively analyze the expression level of *ScoSAD* in the transgenic plants. The results showed that *SAD* expression levels in *ScoSAD*-overexpressing lines T13, T15, and T33 were 7.1, 4.9, and 4.1-fold higher than those in wild-type plants after 12d cold acclimation, respectively (Fig 5). Analysis of variance (ANOVA) showed that expression levels of *ScoSAD* in the T33 and T15 lines were significantly higher than that in the wild type (*P* < 0.05), and that of T13 reached to a very significant level (*P* < 0.01).

### Freeze tolerance was observed in the *ScoSAD* overexpression plants

We next examined whether increasing the expression level of *ScoSAD* can generate the freezing tolerance phenotypes in the transgenic potato plants. Since RFT is a major indicator for freezing tolerance, we therefore examined the RFT values of the transgenic plants at 0 day and 12 days in response to cold treatment. The results showed that the RFT of the T13 and T15 lines was significantly increased when cold treatment for 12 days (*P* < 0.05), and their acclimation capacity reached to 1.0–2.0°C (Table 3). This suggested that the imported *ScoSAD* gene contributed greatly to the enhanced cold acclimation capacity in the potato plants.

**Table 3:** Nonacclimated freezing tolerance (RFT) and acclimation capacity (ΔRFT) of Genetically modified lines and control (WT).

Genotypes	Noacclimated freezing tolerance	Acclimated freezing tolerance	Acclimation capacity
	(RFT in °C)	(RFT in °C)	(ΔRFT in °C)
T13	–3.2±0.3aA	–5.2±0.7aA	2.0
T15	–3.3±0.6 aA	–4.3±1.4abA	1.0
T33	–2.8±0.2 aA	–3.9±0.3abA	1.1
WT	–2.7±1.0 aA	–2.7±0.5bA	0.0

T13-T33, *ScoSAD*-overexpressing lines. WT, wild-type Zhongshu No. 8.

### The profile of fatty acid composition was significantly changed in *ScoSAD* transgenic potato

**Fig 6:**
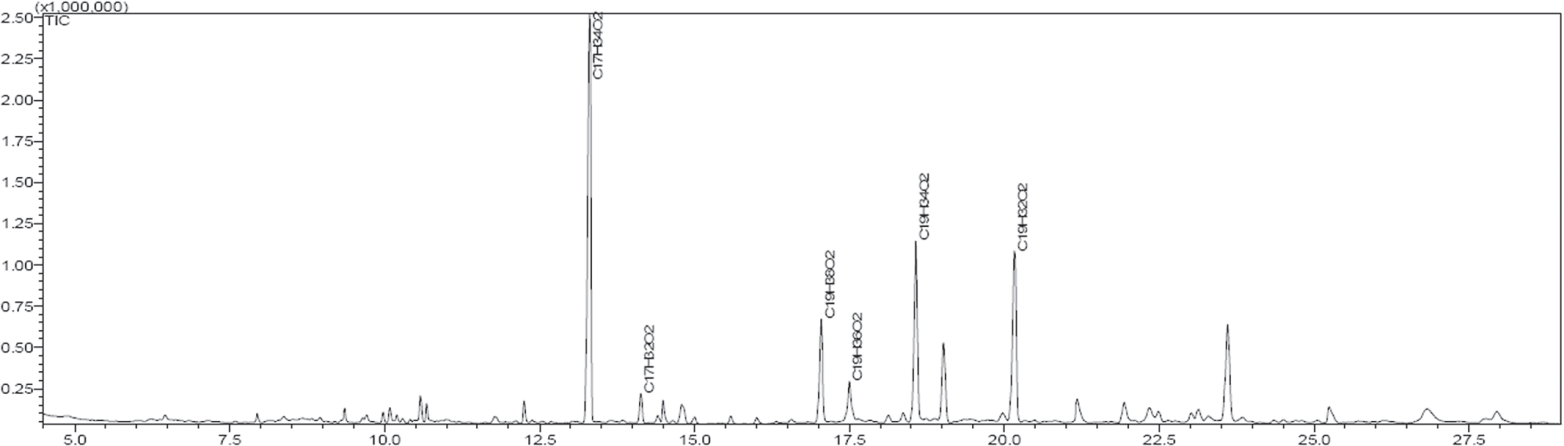
Chromatogram of fatty acid methyl ester products in potato leaves.

Total fatty acids were extracted from mature leaves and methyl esterified for GC analysis (Fig 6). The compositions are listed in Table 4. The T13 line showed a lower palmitic acid (C16:0) content but increased palmitoleic acid (C16:1) content compared to wild-type plants under growth conditions of 20°C day/18°C night (*P* < 0.01). The C16:0 content decreased gradually after 12 days of cold acclimation, whereas that of octadecanoic acid increased slightly. Among the tested lines, only T13 showed a significant change in C16:0 and octadecanoic acid contents after cold acclimation. The oleic acid content was significantly lower in both the T13 and T15 lines after cold acclimation (*P* < 0.05), as well as in T33 (*P* < 0.01). However, the C18:2 content increased highly significant in the three *ScoSAD-*overexpressing lines following cold acclimation, whereas the C18:2 content did not differ in the wild type. Moreover, the C18:3 contents of all three *ScoSAD*-overexpressing lines decreased significantly (P<0.01), whereas no difference was evident in the wild type. This indicates that there may be a causal relationship between C18:3 content and cold acclimation.

**Table 4:** Fatty acid compositions and their relative contents in potato leaves.

Genotype	Fatty acid compositions					
	C16:0	C16:1	C18:0	C18:1	C18:2	C18:3
Cold acclimation 0d						
T13	37.3±2.0abA	0.5±0.1aA	7.6±0.9cB	19.8±1.2abAB	33.9±2.8bAB	0.8±0.1aA
T15	38.4±5.7aA	0.4±0.1abAB	8.7±1.6abcAB	18.3±1.7bcB	33.7±5.7bAB	0.5±0.1bB
T33	37.3±0.8abA	0.5±0.0aA	7.6±0.6cB	19.8±0.8abAB	33.9±1.4bAB	0.8±0.1aA
WT	37.6±1.0abA	0.3±0.1bcAB	8.4±0.4bcAB	20.6±1.2aA	32.6±1.1bB	0.5±0.0bcB
Cold acclimation 12d						
T13	33.3±1.3bA	0.4±0.1aAB	9.9±0.4aA	16.0±0.5dCD	40.1±0.9aA	0.3±0.1dC
T15	34.9±1.2abA	0.5±0.1aA	9.2±0.6abAB	15.5±0.5dC	39.6±1.1aA	0.3±0.0dC
T33	33.7±0.1abA	0.2±0.2cb	7.9±0.3cB	18.03±0.2cBC	39.8±0.3aA	0.4±0.0cdBC
WT	36.7±0.5abA	0.4±0.0abcAB	9.7±0.4abA	19.1±0.1abcAB	33.6±0.5bAB	0.5±0.1bB

## Discussion

Fatty-acid dehydrogenase regulates the unsaturation index of fatty acids to modulate membrane fluidity in response to low temperatures [27]. Soluble SAD catalyzes the formation of the first double bond between carbon atoms 9 and 10 in stearic acid (C18:0) in the plant fatty acid metabolic pathway, generating mono-unsaturated oleic acid (C18:1)[28]. SAD affects the formation of cell membranes and maintains their mobility and semipermeability [11].

*SAD* genes have been cloned from various plants [15, 6, 16, 17, 18]. The multiple sequence alignment showed that the three wild potato species *SAD* genes shared high identity. However, variations at amino acid residues 6, 19, and 21 were detected between the freeze-tolerant *S*. *commersonii* and *S*. *acaule* compared to *S*. *cardiophyllum*, which has no cold acclimation capacity. Moreover, variations at amino acid residues 89, 109, 156, and 202 were detected. These mutated amino acids may affect *SAD* gene expression. Wild potato species SADs always begin with MALN, as determined in this study, but with MALK in woody plants [29]. The phylogenetic analysis showed the three wild potato species have the closest genetic relationship with the *S*.*lycopersicum*, and that its relatives include the *Nicotiana tomentosiformis* and *S*.*tuberosum*.

Few reports on functional characterization of *SAD* genes are available. We performed a functional analysis of the SAD gene, which showed that 12 days of cold acclimation significantly upregulated *SAD* expression in the three *ScoSAD-*overexpressing lines compared with wild-type plants (*P* < 0.05 or < 0.01). This is likely attributable to introduction of exogenous *ScoSAD*, which enhanced SAD dehydrogenase activity and resulted in a high expression level. It also suggests that SAD dehydrogenases are activated by low-temperature stress. The RFT values of the three transgenic lines were not significantly different from that of the wild type, suggesting that *ScoSAD* overexpression does not alter freeze tolerance markedly before cold acclimation. The freeze tolerance of the three transgenic lines was enhanced to different extents after 12 days of cold acclimation, and their RFT values increased by 1–2°C, indicating that *ScoSAD* overexpression enhanced the cold acclimation capacity of the common potato cultivar Zhongshu8. A series of gene responses caused by cold acclimation, It might in turn strengthen the function of *ScoSAD*. In 1995, Kodama et al. [12] reported that the C18:0 content of a SAD gene-mutated *Arabidopsis* was unaffected by low-temperature stress. However, Tasseva et al. [13] showed that upregulation of *SAD* in *Brassica napus* L. under low temperature stress resulted in an increase in C18:0 content. Monica et al. [30] reported that *SAD* overexpression improves cold tolerance in plants, due to enhanced membrane stability caused by increased unsaturated fatty acid content.

Irinaet et al. [31] transformed the *Thermophileccyano bacterium SAD* gene into tobacco and performed a functional analysis. The injury rate of F1 transgenic tobacco progeny was 70% at −5°C for 1 h, whereas that of wild-type plants was 90%. The germination rate of transgenic tobacco seeds was 91% at 0°C for 2 h, whereas that of the wild type was 56%. They concluded that overexpression of the tobacco *SAD* gene increased the unsaturated fatty acid content of the membrane, which improved the freeze tolerance of the transgenic plants and seeds. Therefore, introduction of the *SAD* gene may alter the fatty acid composition of plants.

*SAD* expression promotes conversion of C18:0 to C18:1, providing more substrate for synthesis of di-unsaturated C18:2 and tri-unsaturated C18:3. However, the overexpression of *ScoSAD* in potato plants did not cause remarkable increase in C18:1 of polar lipids, due to a possible further channelling of increased C18:1 towards C18:2 observed in transgene plants. The fatty acid composition analysis showed that the major components were C16:0 and C18:2, followed by C18:1 and C18:0, with a minimal amount of C16:1. These results are consistent with those of Palta et al. [32]. The increase and decrease in linoleic and C18:1 contents were significantly higher than in the wild-type after 12 days cold acclimation, (*P* < 0.05 or *P* < 0.01), suggesting that production of C18:2 is increased when C18:1 is consumed. Yoshida et al. [33] reported that overwintered Mori bark has a significantly higher C18:2 content and lower C18:3 in the membrane. Similar results have been reported in pine needles [8], cranberry leaves [7], and apple [34]. The highest C18:3 fatty acid content detected was in transgenic potato [30], suggesting that C18:2 plays a key role in enhancing the freeze tolerance of potato plants.

The *SAD* genes were cloned from four *Solanum* species, *S*. *commersonii*, *S*. *acaule*, *S*. *cardiophyllum*, and *S*. *tuberosum* (cv Zhongshu8), which differ in their freeze tolerance and cold acclimation. Sequence alignments indicated that 13 amino acid differences in the *SAD* genes of the three wild potato species, which might be associated with the cold acclimation capacity of *S*. *cardiophyllum*. *pBI121* vectors containing *ScoSAD* were constructed and introduced into Zhongshu8 by *A*. *tumefaciens-*mediated transformation. The positive transgenic plants exhibited increased cold acclimation capacity. Fatty acid content analysis showed increased C18:2 content in transgenic plants, indicating that C18:2 plays a key role in the cold acclimation capacity of potato plants. We conclude that potato *SAD* overexpression caused an increase in membrane C18:2 content, which improved the cold acclimation capacity of transgenic potato plants.
